# Surface Polymers on Multiwalled Carbon Nanotubes for Selective Extraction and Electrochemical Determination of Rhodamine B in Food Samples

**DOI:** 10.3390/molecules26092670

**Published:** 2021-05-02

**Authors:** Yassine Benmassaoud, Khaled Murtada, Rachid Salghi, Mohammed Zougagh, Ángel Ríos

**Affiliations:** 1 Department of Analytical Chemistry and Food Technology, University of Castilla-La Mancha, 13071 Ciudad Real, Spain; yassinebenmassaoud@gmail.com (Y.B.); kmurtada@uwaterloo.ca (K.M.); 2 Analytical-NANO-Group, Regional Institute for Applied Chemistry Research (IRICA), 13071 Ciudad Real, Spain; mohammed.zougagh@uclm.es; 3 Laboratory of Applied Chemistry and Environment, Ecole Nationale des Sciences Appliquées (ENSA), Université Ibn Zohr, P.O. Box 1136, Agadir 80000, Morocco; r.salghi@uiz.ac.ma; 4 Department of Analytical Chemistry and Food Technology, Faculty of Pharmacy, University of Castilla-La Mancha, 02071 Albacete, Spain

**Keywords:** Rhodamine B, molecularly imprinted polymer, magnetic solid phase extraction, electrochemical detection, magnetic multiwalled carbon nanotube, screen-printed carbon electrode

## Abstract

In this study, we combine magnetic solid phase extraction (MSPE), with the screen-printed carbon electrode (SPCE) modified by a molecular imprinted polymer (MIP) for sensitive and selective extraction and electrochemical determination of Rhodamine B in food samples. A magnetic solid phase extraction (MSPE) was carried out using magnetic poly(styrene-co-divinylbenzene) (PS-DVB) and magnetic nanoparticles (MNPs) synthetized on the surface of multiwalled carbon nanotubes (MWCNTs). An MIP was prepared on the surface of MWCNTs in the presence of titanium oxide nanoparticles (TiO_2_NPs) modifying the SPCE for the rapid electrochemical detection of Rhodamine B. The MIPs synthesis was optimized by varying the activated titanium oxide (TiO_2_) and multiwalled carbon nanotubes (MWCNTs) amounts. The MSPE and electrochemical detection conditions were optimized as well. The present method exhibited good selectivity, high sensitivity, and good reproducibility towards the determination of Rhodamine B, making it a suitable method for the determination of Rhodamine B in food samples.

## 1. Introduction

Belonging to the Xanthene class, Rhodamine B is a synthetic dye largely used in cosmetic, textile, pharmaceutical, and several other industries. Nevertheless, the potential health consequences on both humans and animals from its use have been proven, leading to its prohibition in the food industry [1].

Several analytical methods have been optimized for the determination of Rhodamine B including chromatographic and electrophoretic techniques, spectroscopic, electrochemical, and fluorometric detection [2,3,4,5,6,7,8]. Each one of these methods has drawbacks for the determination of Rhodamine B in different matrices. Electrochemical detection is an important technique for the determination of Rhodamine B due to its simplicity, fast response, high sensitivity, and cheap instrumentation [9].

Various preconcentration methods were also applied, such as cloud point extraction (CPE) [10], solid phase extraction (SPE) [11], and liquid-liquid extraction (LLE) [12]. The magnetic solid phase extraction (MSPE) technique provides various advantages as a sample preparation and preconcentration alternative: mainly, time preservation, high preconcentration factor, and adsorbent reusability [13]. Functioning with a lock/key system, molecularly imprinted polymers (MIPs) provide the specific and selective binding of the target molecule (template) [14]. They are considered as a synthetic equivalent to natural antibodies and antigens. The production procedure consists of polymerization in the presence of a crosslinking agent and a solvent, after forming a complex between the functional lock/key and the template molecule [14]. The target molecule is then removed, leaving specific compatible cavities. MIPs have been used in many areas due to their high stability, inexpensiveness, and simple preparation process, such as sensors, separation, and catalysis [15,16].

The screen-printing technique is a well-known approach to perform (bio)chemical sensors [17]. The screen-printed electrodes (SPEs) are very adaptable as they could be easily modified using different materials such as nanoparticles and polymers. Apart from that, SPEs are inexpensive, portable, and allow for the analysis of less than 50 µL leading to possible industrial use. Screen-printed carbon electrodes (SPCE) are widely used, especially in environmental and food fields [18].

In the present work, a screening electrochemical method for the determination of Rhodamine B in foodstuffs has been successfully developed. A correspondent MIP was first prepared involving MWCNT and TiO_2_ nanoparticles and used as modifying material for the SPCE. Then, the MSPE method was developed as well using MMWCNT-PS-DVB as an adsorbent. The sensitivity of the developed electrode has been compared with the non-modified SPCE and the TiO_2_-MWCNT-SPCE for determination of Rhodamine B. The results show that the developed electrode has high sensitivity in comparison with the non-modified SPCE and the TiO_2_-MWCNT-SPCE. The electrochemical performance of the developed method is also explained for the determination of Rhodamine B in food samples obtaining high sensitivity, good selectivity, and good stability.

## 2. Results

### 2.1. Characterization of TiO_2_-MWCNT-MIP and MMWCNTs-PS-DVB Composites

**Figure 1:**
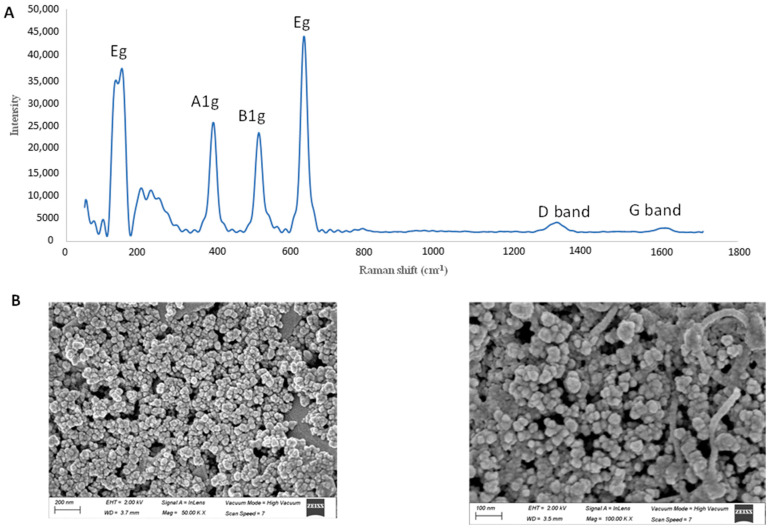
Characterization of TiO_2_-MWCNTs-MIP by Raman spectrum (**A**), and SEM images (**B**), images are presented at magnification of 50K× and 100K×.

The synthesized TiO_2_-MWCNT-MIP composite was characterized by Raman spectrometry (Figure 1A). The registered Raman spectra showed various scattering bands involving the characteristic D (1324 cm^−1^) and G (1605 cm^−1^) band of the MWCNT as well as E_g_ (148 cm^−1^), B_1g_ (397 cm^−1^), A_1g_ (517 cm^−1^), and E_g_ (640 cm^−1^) bands from TiO_2_. SEM technique was used to obtain information about the morphology of TiO_2_-MWCNT-MIP, as shown in Figure 1B. At 50.00 K× magnification, it was possible to observe the formation of homogeneous agglomerates, formed by spherical particles. The detailed view (100.00 K× magnification) clearly reveals the presence of MWCNTs in the TiO_2_-MWCNT-MIP.

To confirm the synthesis of the MMWCNTs-PS-DVB composite, the synthesized material was characterized by FT-IR, Raman spectroscopy, and TEM, available in the Supplementary Material (Figure S1). In the FT-IR spectra, the characteristic bands at ~1700 cm^−1^ of aromatic (C=C) bonding and ~2800 cm^−1^ of alkyl (C-H) stretches resulting from the PS-DVB were observed. A band at ~3085 cm^−1^ corresponding to the aromatic (C=C) stretching and a broad band at ~3400 cm^−1^ indicating the presence of hydroxyl groups onto the surface nanomaterials, were also observed (Figure S1a). In Raman spectra, the characteristic D band at ~1351 cm^−1^ and G band at ~1588 cm^−1^ for MWCNTs were observed in the prepared MMWCNTs-PS-DVB composite, as shown in Figure S1b. The TEM micrograph of the MMWCNTs-PS-DVB composite appeared to show the incorporation of the magnetic nanoparticles onto the surface of MWCNTs and PS-DVB (Figure S1c).

### 2.2. Electrochemical Behavior of Rhodamine B

**Figure 2:**
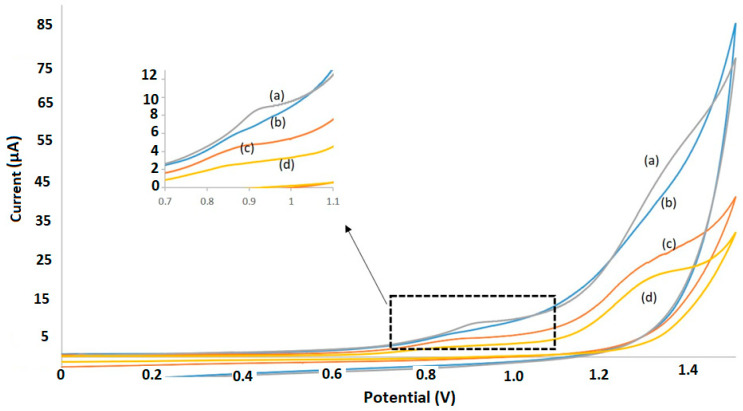
Cyclic voltammograms of different electrodes: (**a**) MIP-SPCE, (**b**) NIP-SPCE, (**c**) MWCNT/TiO_2_-SPCE, and (**d**) non-modified SPCE at 10 mg L^−1^ Rhodamine B in 1 M H_3_PO_4_, at scan rate of 50 mV s^−1^.

The electrochemical behavior of Rhodamine B was tested by comparing different electrodes. Cyclic voltammetry was performed for 10 mg L^−1^ of Rhodamine B using 1 M of H_3_PO_4_ as the supporting electrolyte. The prepared sensor TiO_2_-MWCNT-MIP-SPCE displayed higher sensitivity in comparison with the non-selective SPCE, the TiO_2_-MWCNT-SPCE, and the non-modified SPCE (Figure 2). In addition, the MIP-SPCE offers a high sensitivity compared with the NIP-SPCE (Figure 2b). Thus, it can be said that the MIP-SPCE is very useful for the investigation of the electrochemical behavior of Rhodamine B.

As shown in Figure 2a, the cyclic voltammogram curves obtained for the TiO_2_-MWCNT-MIP-SPCE exhibited an oxidation peak of Rhodamine B, within the potential range tested. It suggests that the oxidation reaction of Rhodamine B on the TiO_2_-MWCNT-MIP-SPCE is totally irreversible. The oxidation current of the TiO_2_-MWCNT-MIP-SPCE (0.9 V) at the TiO_2_-MWCNT-MIP-SPCE was higher than the other compared electrodes (Figure 2). On the basis of the 1H^+^ per two electrons stoichiometry, the electrochemical process of Rhodamine B on the TiO_2_-MWCNT-MIP-SPCE is one H^+^ and two electrons reaction. A possible oxidation mechanism for Rhodamine B is shown in Figure S2. Differential pulse voltammetry (DPV) was also performed at a low concentration of Rhodamine B (100 ng mL^−1^) using 1 M of H_3_PO_4_ as supporting electrolyte (Figure S3).

### 2.3. Optimization of the MSPE Parameters

By analyzing a 10 µg mL^−1^ standard solution of Rhodamine B, several parameters affecting the MSPE were optimized. The pH effect was first investigated in a 1–12 range; the best adsorption result was achieved at pH = 5. The contact time was studied between 1 and 20 min, proving that 7 min were enough for maximum adsorption. The initial volume was fixed at 50 mL after studying a 10–75 mL range. The amount of MMWCNT-PS-DVB was then studied between 50 and 500 mg and 400 mg were sufficient for a maximum adsorption. The desorption part was optimized afterwards; using 2 mL of pure EtOH exhibits better recoveries results than MeOH, ACN and acetone. The eluted solution was then evaporated and reconstituted in an optimal volume of 250 µL of H_3_PO_4_ studied in a 100–500 µL range. After each extraction, MMWCNTs-PS-DVB was washed two times with 2 mL of EtOH, and it was also confirmed that the sorbent can be re-used at least four times with the same extraction efficiency. As well as the extraction efficiency, the adsorbent reusability is a crucial criterium in an extraction process. The reusability of MMWCNT-PS-DVB minimizes the total amount of the waste generation for landfills and reduces the costs of extractions. The extraction efficiencies of the MMWCNT-PS-DVB adsorbent during 15 successive extraction cycles indicated that no significant depletion could be observed after 15 extraction cycles when the MMWCNT-PS-DVB adsorbent was used.

### 2.4. Optimization the Electrochemical Parameters

**Figure 3:**
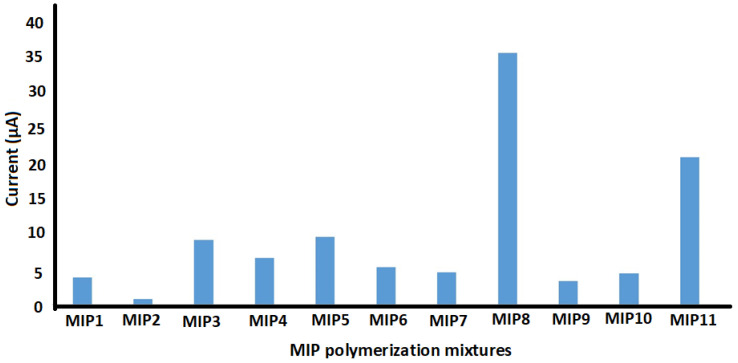
Electrochemical response of 10 mg L^−1^ Rhodamine B in 1 M H_3_PO_4_ using 11 distinct MIPs as electrochemical film modifiers for SPCE.

Eleven different MIPs were synthesized with different amounts of activated TiO_2_, MWCNT, Rhodamine B, functional monomer AM, and KH550 as summarized in Table 1. The resulting polymers showed distinct responses while applied as electrochemical film modifiers for SPCE in the detection of Rhodamine B. In fact, MIP8 provided the best sensitivity (Figure 3).

**Figure 4:**
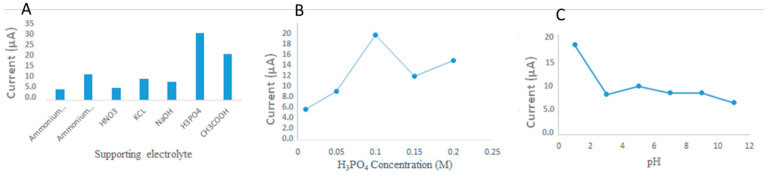
Analytical signals obtained for detection of Rhodamine B: (**A**) different supporting electrolytes, (**B**) different H_3_PO_4_ concentrations, and (**C**) pH (1, 3, 5, 7, 9, and 11).

The parameters affecting the electrochemical detection of Rhodamine B were then optimized, starting with the electrode modification volume by testing 2, 4, 6, 8, and 10 µL. The best results were achieved with 6 µL. Several electrolyte solutions were then tested, namely, ammonium acetate, ammonium phosphate, HNO_3_, KCl, NaOH, H_3_PO_4_, and CH_3_COOH (0.1 M of each). The best detection was provided using 0.1 M of H_3_PO_4_ (Figure 4). The effects of adjusting the pH value of the supporting electrolyte on the oxidative behavior of Rhodamine B disclose significant information about the mechanisms of the electrochemical reactions at the modified electrode. Therefore, the influences of pH and the supporting electrolyte on the electrooxidation of Rhodamine B were carefully investigated by linear sweep voltammetry. The suitable pH conditions were preliminarily studied using H_3_PO_4_ at different pH values as representatives of acidic, neutral, and basic buffer electrolytes, respectively. The obtained voltammograms from the simultaneous detection are shown in Figure 4. With increasing pH value, the anodic currents of Rhodamine B decreased obviously. We believe that in our case, an acidic supporting electrolyte would strongly increase the current. By considering the pKa values of Rhodamine B, the results can be attributed to the fact that the ionic forms of the target analytes affect the electrooxidation processes at the electrode surface. These ionic forms in neutral and basic supporting electrolytes transfer almost no electrons at the electrode surface. From these results, it can be concluded that an acidic supporting electrolyte is suitable for the detection of Rhodamine B. Finally, the detected volume was studied in the range of 10–50 µL, concluding 40 µL as the ideal droplet volume.

The influence of foreign compounds in the determination of Rhodamine B was investigated for various and specific interfering substances in the samples, including Rhodamine 123, Rhodamine 6G (5 times content), and ascorbic acid, citric acid, glucose, maltose, Mg^+2^, Cu^+2^, and Fe^+2^ (20 times content). The possibility of interference was considered when the analytical variation of >5% compared to the signal obtained with the proposed method in the absence of the interfering compounds. The interferences results exhibited that no interferences occurred for the interference analyte ratios (Table 2).

### 2.5. Analytical Application

**Figure 5:**
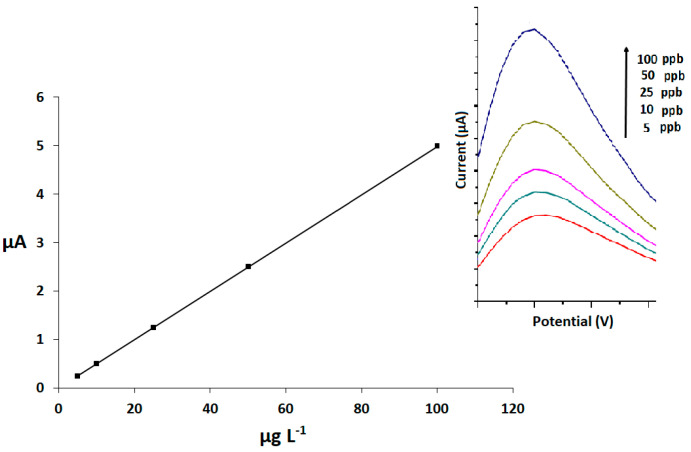
Calibration curve and DPV results at different concentration in the range 5—100 ng mL^-1^.

The analytical performances of the proposed method were settled under the optimized conditions, using differential pulse voltammetry (DPV) as a detection technique. The calibration curve was plotted within a concentration range of 5–100 ng mL^−1^ (Figure 5). The precision was investigated by the analysis of 10 series of 10 replicates of 25 ng mL^−1^ of Rhodamine B standard solution; the relative standard solution calculated was 6.59%. The limit of detection (LOD) and limit of quantification (LOQ) were 1.44 and 4.81 ng mL^−1^, respectively. The figure of merit of the present method is summarized in Table 3. As can be seen in this table, the detection limit of the MIP/SPCE-MSPE method was found to be 1.44 μg L^−1^, which is better than the other methods for detection of Rhodamine B. Thus, it can be said that the MIP/SPCE-MSPE method is very useful for investigation of the electrochemical behavior of Rhodamine B and its determination; SPCE possesses important advantages such as high electrochemical reactivity, commercial availability, good mechanical rigidity, low cost, low technology, ease of modification, and renewable surface. The developed study provides good sensitivity in comparison with methods previously reported for the determination of Rhodamine B (Table 4). The applicability of the described method was investigated by the analysis of Rhodamine B in two types of samples—chili powder and tomato sauce extracts—which were found to contain no Rhodamine B. Thus, these food samples were spiked with distinct concentrations of Rhodamine B that were calculated using the calibration equation. The obtained results are presented in Table 5.

## 3. Materials and Methods

### 3.1. Reagents, Standards, and Samples

Titanium (IV) oxide (TiO_2_, anatase, 20–30 nm particle size), (3-Aminopropyl) triethoxysilane (KH-550, >98%), Rhodamine B, Rhodamine 6G, Rhodamine 123, acrylamide (AM, >99%), ethylene glycol dimethacrylate (EDMA), 2,2’-Azobisisobutyronile (AIBN), styrene (≥99%), divinylbenzene (DVB) (80%), magnesium sulfate (99%), copper (II) sulfate (>99%), iron (II) chloride tetrahydrate (>98%), iron (III) chloride hexahydrate (>97%), ethylene glycol (98%), ammonium acetate (98%), ammonium phosphate (>99%), phosphoric acid (>85%) were purchased from Sigma-Aldrich (St. Louis, MO, USA). Acetic acid (>99.7%), nitric acid (HNO_3_, >69%), ascorbic acid (>98%), citric acid (>99%), glucose (>99%), maltose monohydrate (>99%) were obtained from Panreac (Barcelona, Spain). Methanol (MeOH), ethanol (EtOH), acetonitrile (ACN) and acetone were delivered by Fisher Scientific (Geel, Belgium). MWCNTs of 30 ± 15 nm diameter (95% purity) were obtained from NanoLab Inc. (Waltham, MA, USA). Purified water with a Milli-Q system (Millipore, Bedford, MA, USA) was used for all the preparations.

### 3.2. Apparatus and Instruments

Electrochemical detection was carried out on a CH Instruments Model 800D Series (Austin, Texas, USA). All the experiments were carried out using a screen-printed carbon electrodes (SPCEs) system (Dropsens, Oviedo, Spain).

Transmission electron microscopy (TEM) images were gathered with a Jeol JEM 2011 microscope operating at 200 kV and an Orius Digital Camera (2 × 2 MegaPixel) from Gatan (Pleasanton, CA, USA). For Fourier transform infrared spectroscopy (FTIR), model FT/IR 4200 (Jasco, Tokyo, Japan) was utilized. Raman measurements were performed with a portable Raman Spectrometer system (B&W TEK Inc., Newark, DE, USA) at a wavelength of 785 nm.

### 3.3. Synthesis of Molecularly Imprinted Polymer (MIP)

Firstly, 0.5 g of MWCNT were treated in a 50 mL mixture of 1:3 (*v*:*v*) HNO_3_:H_2_SO_4_ by refluxing for 8 h. The resulting mixture was then diluted in deionized water and centrifugated for 15 min various times until the supernatant pH outtopped 3. The remaining functionalized MWCNT was dried overnight at 80 °C.

The activation of TiO_2_ nanoparticles was achieved then by dispersing 3 g in 15 mL of HNO_3_ (69%), stirring for 24 h at room temperature followed by vacuum drying at 60 °C.

The functionalized MWCNT and the activated TiO_2_ were mixed and the pH was adjusted to pH = 4 using 5 wt% acetic acid. Then, KH-550 was added and the solution was mixed for 6 h at 80 °C. The resulting product was separated by centrifugation, washed by distilled water and MeOH, and vacuum dried at 60 °C for 24 h.

**Figure 6:**
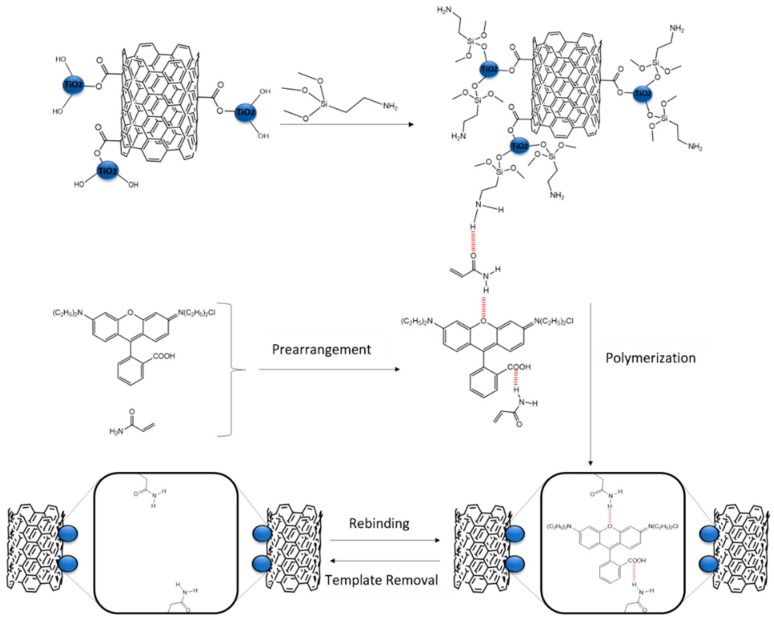
Schematic illustration of the construction process of MIP.

Both Rhodamine B and the functional monomer AM were dissolved in ACN by stirring for 8 h. The previously modified MWCNT-TiO_2_ nanoparticles were added to the solution with EDMA as a cross-linking agent and AIBN as an initiator. The mixture was stirred for 6 h at room temperature, purged for 20 min with nitrogen and vacuum dried. Then, the polymerization took place in a water bath for 48 h at 60 °C. After that, the template was removed by washing the product with a mixture of MeOH and acetic acid (95:5, *v*:*v*). Finally, the TiO_2_-MWCNT-MIP was obtained by centrifugation and drying. A non-selective polymer was also synthesized in the absence of Rhodamine B for a verification aim. An anterior fabrication method was used for the adsorption and degradation of Rhodamine B [16]. The stepwise construction process of MIP is illustrated in Figure 6.

### 3.4. Sensor Fabrication

MIPs were ultrasonically dispersed in pure water (with 0.5% Nafion, *v*:*v*), and the concentrations of 1 mg mL^−1^ were obtained individually. The SPCE forming a layer of thin films from nafion-solubilized MIP are more uniformly distributed than those casted by organic solvents [21,22]. Then, 6 µL of the dispersed MIP was casted onto the surface of the SPCE. After drying the modified SPCE by using infrared light lamp for 10 min, the electrode was ready for using.

### 3.5. Sample Preparation

Two grams of chili powder were added to 25 mL of ACN with ultrasonic vibrating for 30 min at room temperature. To recuperate the extract, the solution was centrifuged for 15 min at 10,000 rpm. The extract was then mixed with 2× *g* of sodium chloride and 5 mL of distilled water, then stored at −20 °C for 2 h. Finally, the solution was collected, filtrated, and evaporated overnight under nitrogen gas, and reconstituted with distilled water at pH = 5. The tomato sauce extract was obtained with direct filtration.

### 3.6. Extraction of Rhodamine B by Magnetic Multiwalled Carbon Nanotubes Poly(styrene-co-divinylbenzene) Composite (MMWCNTs-PS-DVB)

The MMWCNT-PS-DVB composite was prepared according to our previous works [23,24,25] and is available in the Supplementary Materials (Section S1.1). 500 mg of the magnetic composite was put into a glass vial, conditioned with (3 × 3) mL of ethanol, and then with deionized water. The supernatant was then separated with a magnet, discarded, and 50 mL of standard solutions of the Rhodamine B or of spiked food samples, previously adjusted to pH = 5, were added into the conical flask. The mixture was stirred at room temperature for 7 min to create a homogenous dispersion and the magnetic polymer containing the adsorbed Rhodamine B was rapidly removed from the solution by applying an external magnetic field, and the supernatant was discarded. The Rhodamine B was eluted by rinsing with 2.0 mL of methanol, and the eluent dried under a stream of N_2_ gas at room temperature. The residue was finally dissolved in 250 µL of H_3_PO_4_ (1 mg mL^−1^, pH = 5) and 40 μL was casted onto the SPCE surface for electrochemical analysis.

## 4. Conclusions

This work reports the electrochemical detection of Rhodamine B by the MIP modified SPCE, assisted via MSPE using MMWCNTs-PS-DVB as an adsorbent. The developed electrode (MIP-SPCE) presented higher sensitivity in comparison with the bare SPCE, and the TiO_2_-MWCNTs-SPCE. The developed method shows high sensitivity and selectivity towards Rhodamine B with a detection limit of 1.44 µg L^−1^. The use of the developed electrode (MIP-SPCE) allows for the implementation of a simple, effective, and rapid method for electrochemical detection of Rhodamine B in food samples. This modification also introduces higher stability and appropriated selectivity, and the simplicity of the method allows it to be used as a rapid screening test for Rhodamine B. On the other hand, some limitations of the use of MIP-SPCE include a short electrode lifetime, poor inter-electrode reproducibility, and not being user-friendly.

## Figures and Tables

**Table 1:** Composition of the polymerization mixtures used for the MIP synthesis.

Polymer	TiO_2_ NPs (mg)	MWCNT (mg)	KH550 (mL)	AM (mg)	Rhodamine B (mg)
MIP1	2	5	4	1.4	2
MIP2	7	5	4	1.4	2
MIP3	20	5	4	1.4	2
MIP4	7	10	4	1.4	2
MIP5	7	15	4	1.4	2
MIP6	7	5	10	1.4	2
MIP7	7	5	1	1.4	2
MIP8	7	5	4	5	2
MIP9	7	5	4	10	2
MIP10	7	5	4	1.4	5
MIP11	7	5	4	1.4	10

MIP: Molecular imprinted polymer, MWCNT: multiwalled carbon nanotube, AM: acrylamide, and KH550: (3-Aminopropyl) triethoxysilane.

**Table 2:** Effect of the presence of potentially interfering compounds in the electrochemical response of Rhodamine B.

Interferent Species	Tolerated Interferent Analyte (*w*/*w*) Ratio ^a^
Rhodamine 123, Rhodamine 6G	>5 ^b^
Ascorbic acid, citric acid, glucose, maltose, Mg^+2^, Cu^+2^, Fe^+2^	>20 ^b^

^a^ 25 µg L^−1^ Rhodamine B. ^b^ Maximum ratio tested.

**Table 3:** Analytical parameters obtained for the Rhodamine B determination.

Analytical Parameter	Rhodamine B
Linear range (µg L^−1^)	5—100
Calibration graph	
Correlation coefficient	0.9979
Intercept	2.253 × 10^−7^
Slope (µg L^−1^)	0.23
Detection limit (µg L^−1^)	1.44
Quantification limit (µg L^−1^)	4.81
RSD (%) (*n* = 10) ^1^	6.59

^1^ 25 µg L^−1^ Rhodamine B.

**Table 4:** Comparison of the proposed method for determination of Rhodamine B with other reported methods.

Method	Linear Range (μg L^−1^)	LOD (μg L^−1^)	Sample Matrix	Recovery (%)	Reference
UV-visible spectrometry-SPE	250–3000	3.14	Soft drink, wastewater and lipstick	96–118	[11]
UV-visible spectrometry-DLLME	100–3000	2.1	Drug, ink, food, cosmetic product, and waste waters	89–101	[12]
Voltammetric-GCE	4.78–956.1	2.93	Fruit juice and preserved fruit	95.5–104	[19]
MIP-SPE-HPLC	100–8000	3.4	Dyed pink melon seeds, pepper and candied purple potato	78.47–101.6	[20]
MIP/SPCE-MSPE	5–100	1.44	Chili powder and tomato sauce	91–97	This work

GCE: glassy carbon electrode, SPE: solid phase extraction, DLLME: dispersive liquid–liquid microextraction, and HPLC: high-performance liquid chromatography.

**Table 5:** Determination results and recovery study of Rhodamine B in food samples by the proposed method.

Sample	Rhodamine B		
	Added (µg kg^−1^)	Found (µg kg^−1^)	Recovery (%), *n* = 3
Chili powder	10	9.38 ± 0.91	93
	25	22.87 ± 1.67	91
	100	95.67 ± 5.67	95.6
Tomato sauce	10	9.56 ± 1.24	95.6
	50	48.45 ± 6.78	97
	100	91.60 ± 4.12	91.6

## Data Availability

The data presented in this study are available in this article.

## Supplementary Materials

The following are available online. The file of supplementary information is available online and contains the following information: Section S1.1: Synthesis of MMWCNT-PS-DVB composite, Figure S1: Characterization of the MMWCNT-PS-DVB composite by means of (a) FTIR spectra, (b) Raman spectra, and (c) TEM image.

## Sample Availability

Samples of the compounds are not available from the authors.

## References

[1] Jain R., Mathur M., Sikarwar S., Mittal A. (2007). Removal of the hazardous dye rhodamine B through photocatalytic and adsorption treatments. J. Environ. Manag..

[2] Qi P., Lin Z., Li J., Wang C., Meng W., Hong H., Zhang X. (2014). Development of a rapid, simple and sensitive HPLC-FLD method for determination of rhodamine B in chili-containing products. Food Chem..

[3] Gagliardi L., De Orsi D., Cavazzutti G., Multari G., Tonelli D. (1996). HPLC determination of rhodamine B (C.I. 45170) in cosmetic products. Chromatographia.

[4] Tatebe C., Zhong X., Ohtsuki T., Kubota H., Sato K., Akiyama H. (2014). A simple and rapid chromatographic method to determine unauthorized basic colorants (rhodamine B, auramine O, and pararosaniline) in processed foods. Food Sci. Nutr..

[5] Chiang T.-L., Wang Y.-C., Ding W.-H. (2012). Trace Determination of Rhodamine B and Rhodamine 6G Dyes in Aqueous Samples by Solid-phase Extraction and High-performance Liquid Chromatography Coupled with Fluorescence Detection. J. Chin. Chem. Soc..

[6] Dongre C., Dekker R., Hoekstra H.J.W.M., Pollnau M., Martinez-Vazquez R., Osellame R., Cerullo G., Ramponi R., Van Weeghel R., Besselink G.A.J. (2008). Fluorescence monitoring of microchip capillary electrophoresis separation with monolithically integrated waveguides. Opt. Lett..

[7] Park M., Bahng S.-H., Woo N., Kang S.H. (2016). Highly sensitive wavelength-dependent nonaqueous capillary electrophoresis for simultaneous screening of various synthetic organic dyes. Talanta.

[8] Xu Y., Wang J., Yao L. (2006). Dating the writing age of black roller and gel inks by gas chromatography and UV–vis spectrophotometer. Forensic Sci. Int..

[9] Murtada K., Moreno V. (2020). Nanomaterials-based electrochemical sensors for the detection of aroma compounds-towards analytical approach. J. Electroanal. Chem..

[10] Pourreza N., Rastegarzadeh S., Larki A. (2008). Micelle-mediated cloud point extraction and spectrophotometric determination of rhodamine B using Triton X-100. Talanta.

[11] Soylak M., Unsal Y.E., Yilmaz E., Tuzen M. (2011). Determination of rhodamine B in soft drink, waste water and lipstick samples after solid phase extraction. Food Chem. Toxicol..

[12] Unsal Y.E., Soylak M., Tuzen M. (2014). Dispersive liquid–liquid microextraction–spectrophotometry combination for determination of rhodamine B in food, water, and environmental samples. Desalin. Water Treat..

[13] Yan J., Cen J.-M., Tan X.-C., Tan S.-F., Wu Y.-Y., Zhang H., Wang Q. (2017). Determination of trace rhodamine B by spectrofluorometry and magnetic solid phase extraction based on a 3D reduced graphene oxide composite. Anal. Methods.

[14] BelBruno J.J. (2019). Molecularly Imprinted Polymers. Chem. Rev..

[15] Bouri M., Lerma-García M.J., Salghi R., Zougagh M., Ríos A. (2012). Selective extraction and determination of catecholamines in urine samples by using a dopamine magnetic molecularly imprinted polymer and capillary electrophoresis. Talanta.

[16] Bao L., Meng M., Sun K., Li W., Zhao D., Li H., He M. (2014). Selective adsorption and degradation of rhodamine B with modified titanium dioxide photocatalyst. J. Appl. Polym. Sci..

[17] Goldberg H.D., Brown R.B., Liu D.P., Meyerhoff M.E. (1994). Screen printing: A technology for the batch fabrication of integrated chemical-sensor arrays. Sens. Actuators B Chem..

[18] Moreno V., Llorent-Martínez E.J., Zougagh M., Ríos A. (2016). Decoration of multi-walled carbon nanotubes with metal nanoparticles in supercritical carbon dioxide medium as a novel approach for the modification of screen-printed electrodes. Talanta.

[19] Yu L., Mao Y., Qu L. (2013). Simple Voltammetric Determination of Rhodamine B by Using the Glassy Carbon Electrode in Fruit Juice and Preserved Fruit. Food Anal. Methods.

[20] Su X., Li X., Li J., Liu M., Lei F., Tan X., Li P., Luo W. (2015). Synthesis and characterization of core–shell magnetic molecularly imprinted polymers for solid-phase extraction and determination of Rhodamine B in food. Food Chem..

[21] Murtada K., Jodeh S., Zougagh M., Ríos Á. (2018). Development of an Aluminium Doped TiO_2_ Nanoparticles-modified Screen Printed Carbon Electrode for Electrochemical Sensing of Vanillin in Food Samples. Electroanalysis.

[22] Murtada K., Salghi R., Ríos Á., Zougagh M. (2020). A sensitive electrochemical sensor based on aluminium doped copper selenide nanoparticles-modified screen printed carbon electrode for determination of L-tyrosine in pharmaceutical samples. J. Electroanal. Chem..

[23] Murtada K., De Andrés F., Ríos Á., Zougagh M. (2018). Determination of antidepressants in human urine extracted by magnetic multiwalled carbon nanotube poly(styrene-co-divinylbenzene) composites and separation by capillary electrophoresis. Electrophoresis.

[24] Murtada K., De Andrés F., Galván I., Ríos Á., Zougagh M. (2020). LC-MS determination of catecholamines and related metabolites in red deer urine and hair extracted using magnetic multi-walled carbon nanotube poly(styrene-co-divinylbenzene) composite. J. Chromatogr. B.

[25] Adelantado C., Murtada K., Ríos Á., Zougagh M. (2018). Magnetic multi-walled carbon nanotube poly(styrene-co-divinylbenzene) for propranolol extraction and separation by capillary electrophoresis. Bioanalysis.

