# Supplementary material for: Surface Polymers on Multiwalled Carbon Nanotubes for Selective Extraction and Electrochemical Determination of Rhodamine B in Food Samples
Source: Molecules. 2021 May 2;26(9):2670. doi: 10.3390/molecules26092670 (PMC8124413; doi:10.3390/molecules26092670)
Supplement: Supplementary file 1 [file molecules-26-02670-s001.zip › molecules-1146302-supplementary.pdf]

Supplementary Material

Surface Polymers on Multiwalled Carbon Nanotubes for Selective Extraction and Electrochemical Determination of Rhodamine B in Food Samples

Yassine Benmassaoud [1,2,3], Khaled Murtada [1,2,‡], Rachid Salghi [3], Mohammed Zougagh [2,4], Ángel Ríos [1,2,*]

[1] Department of Analytical Chemistry and Food Technology, University of Castilla-La Mancha, Ciudad Real 13071, Spain.
[2] Regional Institute for Applied Chemistry Research (IRICA), Ciudad Real 13071, Spain.
[3] Laboratory of Applied Chemistry and Environment, ENSA, Université Ibn Zohr, PO Box 1136, 80000 Agadir, Morocco.
[4] Department of Analytical Chemistry and Food Technology, Faculty of Pharmacy, University of Castilla-La Mancha, Albacete 02071, Spain

*Corresponding author: Angel.Rios@uclm.es (A. Rios)

‡ Current address: Department of Chemistry, University of Waterloo, 200 University Avenue West, Waterloo, Ontario, N2L 3G1, Canada.

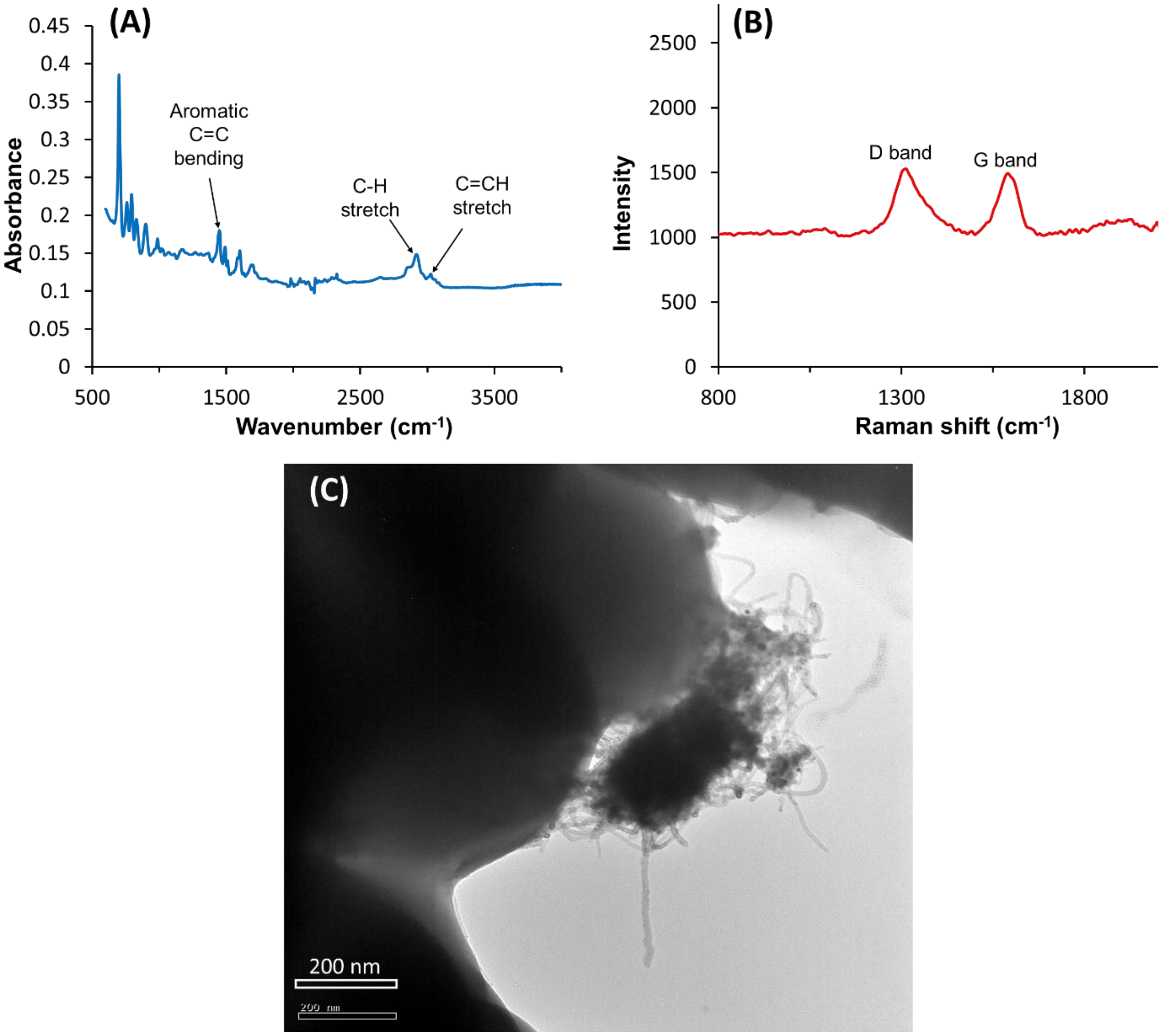

Figure S1. Characterization of the MMWCNT-PS-DVB composite by means of (a) FTIR spectra, (b) Raman spectra, and (c) TEM image.

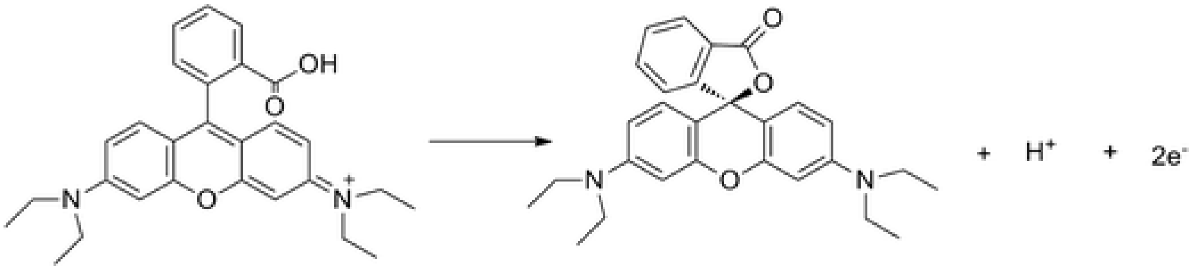

Figure S2. Oxidation mechanism of Rhodamine B at the TiO_2-MWCNT-MIP-SPCE.

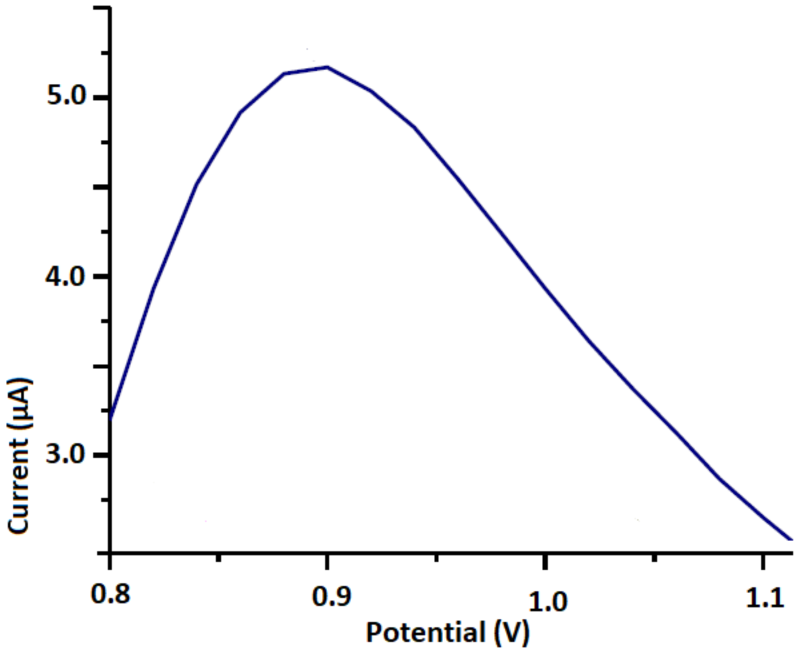

Figure S3. Differential pulse voltammetry (DPV) of TiO_2-MWCNT-MIP-SPCE modified electrode, at 100 ng mL^{-1} Rhodamine B in 1 M H_3PO_4.

Section S1.1: Synthesis of MMWCNT-PS-DVB composite

Firstly, magnetic multi-walled carbon nanotubes (MMWCNTs) were prepared by thermal decomposition of iron(III) magnetic precursor and MWCNTs. Briefly, this hydrothermal synthesis includes the mixture of 140 mg of iron(III) chloride hexahydrate and 40 mg of MWCNTs, which was then followed by its suspension in 7.5 mL of ethylene glycol in a 25 mL glass bottle. Later, 0.36 g of sodium acetate was added to the mixture, and the resulting mixture was sonicated for 10 min and kept at room temperature for 1 h. After that, the glass bottle was heated to high temperature (up to 200 °C) for 48 h to complete the reaction and, it was then cooled slowly to room temperature. The product can then be collected and washed with distilled water. Then, an external magnet was applied for the separation of the MMWCNTs, and the nanomaterial obtained was dried at 70 °C and stored at room temperature.

After then, MMWCNT-PS-DVB composite was synthesized by mixing 9.79 mmol of DVB, 23.69 mmol of styrene, 0.36 g of AIBN, 0.15 g of MMWCNTs and 25 mL of ACN for 24 h at 70 °C. After sonication (5 min), the reaction mixture was purged with nitrogen gas (10 min) and sealed. The final product was collected, thoroughly washed three times with water and finally stored for 24 h in a vacuum desiccator.
